# Supplementary material for: Normal & reversed spin mobility in a diradical by electron-vibration coupling
Source: Nat Commun. 2021 Oct 29;12:6262. doi: 10.1038/s41467-021-26368-8 (PMC8556253; doi:10.1038/s41467-021-26368-8)
Supplement: Supplementary file 1 — Supplementary Information [file 41467_2021_26368_MOESM1_ESM.pdf]

Supplementary Information for:

Normal & Reversed Spin Mobility in a Diradical By Electron-Vibration Coupling

Yi Shen,[a] Guodong Xue,[a] Yasi Dai,[b] Sergio Moles Quintero,[c] Hanjiao Chen,[d] Dongsheng Wang,[a] Fang Miao,[a]* Fabrizia Negri,[b]* Yonghao Zheng,[a]* Juan Casado[c]*

[a] School of Optoelectronic Science and Engineering, University of Electronic Science and Technology of China (UESTC) Chengdu 610054, People's Republic of China.

[b] Università di Bologna, Dipartimento di Chimica 'Giacomo Ciamician', Via F. Selmi, 2, 40126 Bologna, Italy and INSTM, UdR Bologna, Italy.

[c] Department of Physical Chemistry, University of Málaga, Campus de Teatinos s/n, Málaga 29071, Spain.

[d] Analytical & Testing Center, Sichuan University, Chengdu 610064, People's Republic of China.

E-mail addresses: zhengyonghao@uestc.edu.cn; casado@uma.es; fabrizia.negri@unibo.it; miaofang@uestc.edu.cn

1. Methods and Materials

All reagents were purchased from Sigma-Aldrich, Alfa, Acros and Adamas and used as received. Flash column chromatography was performed with Haiyang silica gel (200-300 mesh), and Greagent neutral Aluminum Oxide (200-300 mesh). tetrahydrofuran (THF) was freshly distilled from Na under N_2. Anhydrous Na_2SO_4 was used for drying organic extracts, and all volatiles were removed under reduced pressure. All reaction mixtures and column eluents were monitored by TLC using commercial Huanghai glass plates (HSGF 254, 2.5 x 8 cm). The plates were visualized under UV radiation at 254 and 365 nm. UV absorption spectra were recorded on a Shimadzu UV-2600 UV-VIS spectrophotometer in spectroscopy grade dichloromethane (DCM). NMR spectra were obtained on a Bruker AV II-400 MHz. MALDI-TOF mass spectra (MS) were recorded on a SHIMADZU iD plus Performance using anthracene-1, 8, 9-triol as matrix. ESR measurements were carried out on a Bruker EMX plus X-band spectrometer with 9.8 GHz microwave frequency. High resolution mass spectra (HRMS) were measured on a Waters-Q-TOF-Premier (ESI). SQUID measurements were carried out on a Quantum Design (MPMS-SQUID VSM-094). For the variable temperature ESR, the ESR intensity (I) increased with increasing temperature (T), and the data was fitted by modified Bleaney-Bowers equation. Thermogravimetric analysis (TGA) measurements were performed on NETZSCH TG 209F1 Iris thermal gravimetric analyzer. Elemental analysis measurements were performed on a Leeman Labs Euro EA 3000 elemental analyzer. Cyclic voltammograms were measured on a Shanghai Chenhua CHI 660E electrochemical workstation. A gold disk was used as working electrode, platinum wire was used as counter electrode, and Ag/AgCl (3 M KCl solution) was used as reference electrode.

2. Synthetic details

Synthesis of CARH

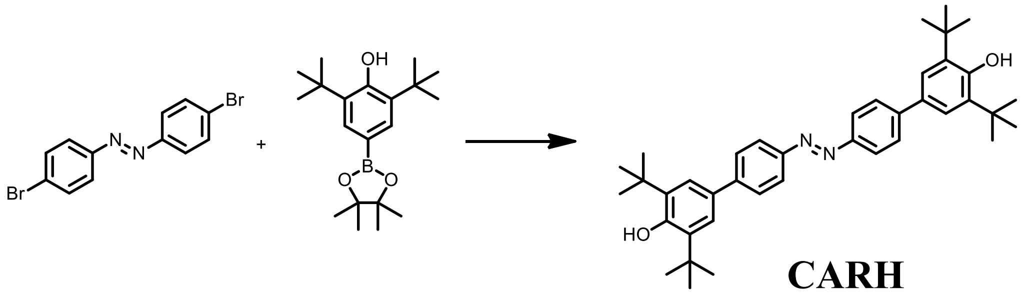

CARH

4,4'-Dibromoazobenzene (200 mg, 1.0 eq), 2,6-di-tert-butyl-4-(4,4,5,5-tetramethyl-1,3,2-dioxaborolan-2-yl)phenol (410 mg, 2.2 eq), Na_2CO_3 (230 mg, 6.0 eq) and Tetrakis(triphenylphosphine) palladium(0) (70 mg, 10% mmol) were suspended in a mixture of THF (30 ml) and water (12 ml) under an atmosphere of nitrogen. After stirring the reaction mixture at 90 °C for 14 hours, the suspension was cooled down to room temperature. Subsequently, the crude product was extracted with DCM. The combined organic layers were washed with water, dried over Na_2SO_4 and the solvent was removed under reduced pressure. The crude product was purified by silica gel column chromatography (hexane/ dichloromethane, 5:1) to yield **CARH** as a yellow solid.

Yield: 89 % (225 mg). ^{1}H NMR (400 MHz $CDCl_3$): δ = 7.99 (4 H, d, J_{HH} = 8 Hz), 7.70 (4 H, d, J_{HH} = 8 Hz), 7.49 (4 H, s), 5.34 (2 H, s), 1.52(CH_3, 36 H, s). ^{13}C NMR (100 MHz, $CDCl_3$): δ = 30.34, 34.53, 123.28, 124.08, 127.42, 131.50, 136.39, 144.61, 151.39, 154.09. HRM(ESR$^+$): calcd for $C_{40}H_{50}N_2O_2$ 590.3872, found $[M+H]^+$ 591.3976. UV/vis/NIR (DCM) λ_{max} (ε): trans- 252(36814), 391(65781) cis-259(36224), 386(26607).

Synthesis of diradical CAR

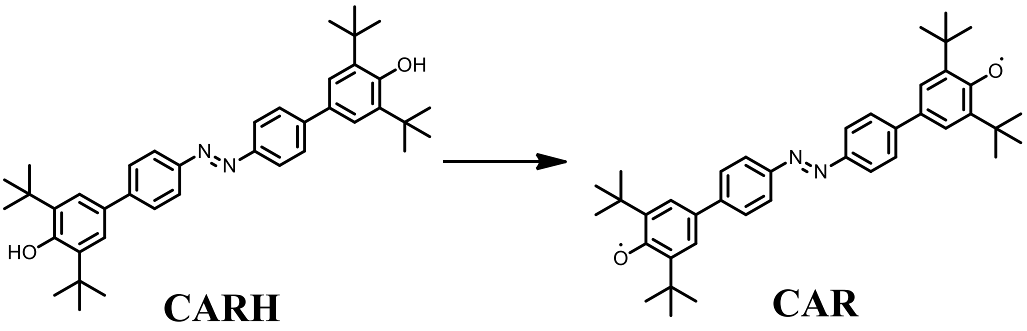

CARH **CAR**

Lead (IV) oxide (232 mg, 40.0 eq) and **CARH** (50 mg, 1.0 eq) were suspended in DCM (10 mL). After stirring the reaction mixture at room temperature for 30 minutes, the excess lead(IV) oxide was filtered off and the solvent was removed under reduced pressure to yield **CAR** as a black solid. Yield: 95 %. HRM(ESR$^+$): calcd for $C_{40}H_{48}N_2O_2$ 588.3716, found $[M+H]^+$ 589.3748. UV/vis/NIR (DCM) λ_{max} (ε): 295(15264), 368(39424), 439(24424), 640(147350).

3. NMR spectra and TGA data.

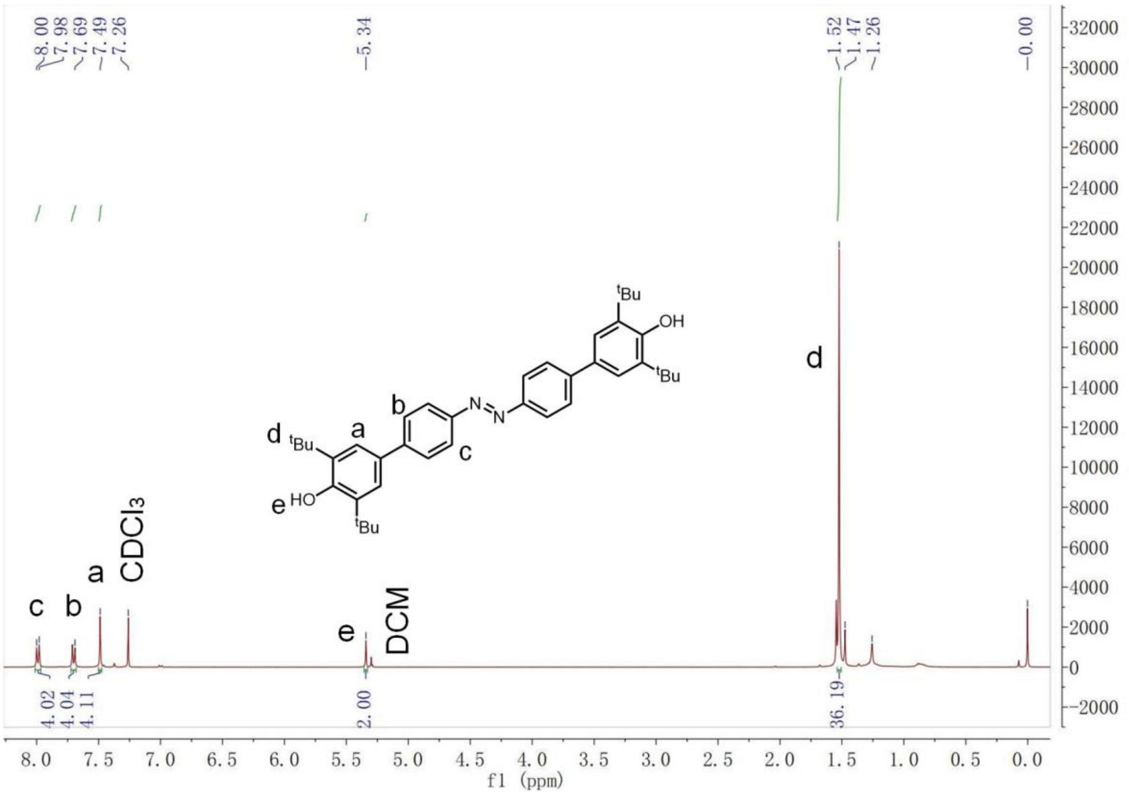

Figure S1. ^{1}H NMR spectrum of **CARH** in CD_3Cl.

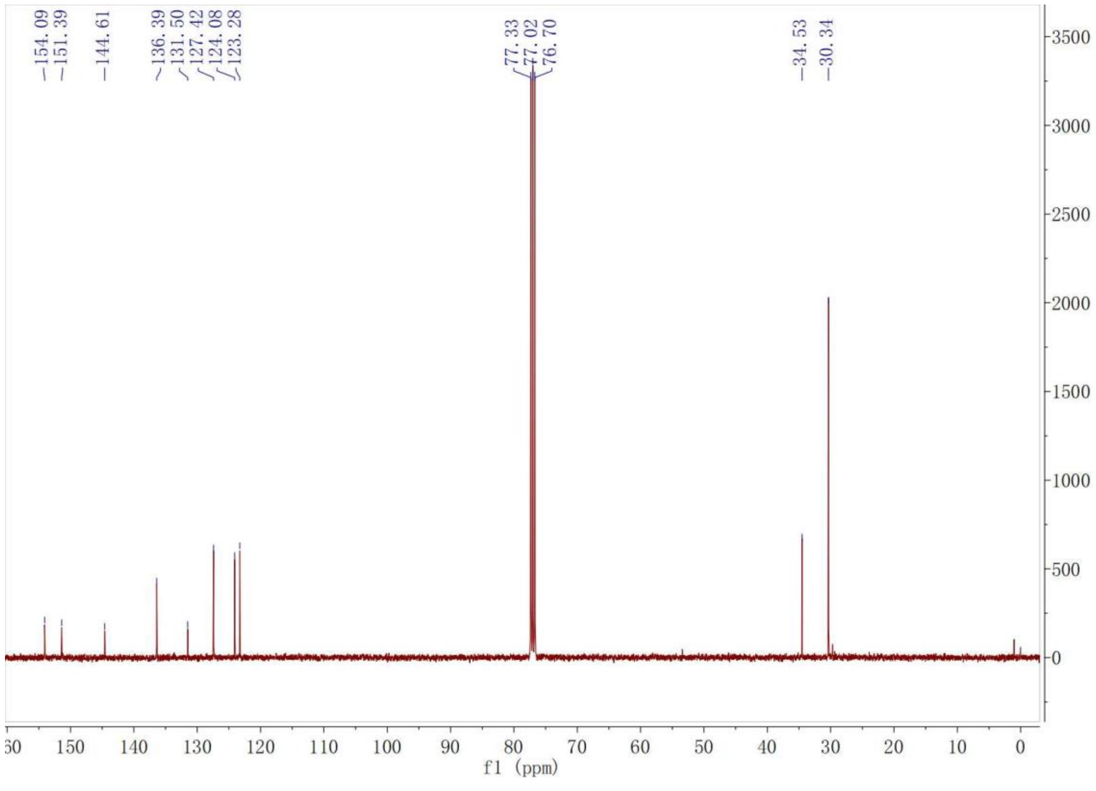

Figure S2. ^{13}C NMR spectrum of **CARH** in CD_3Cl.

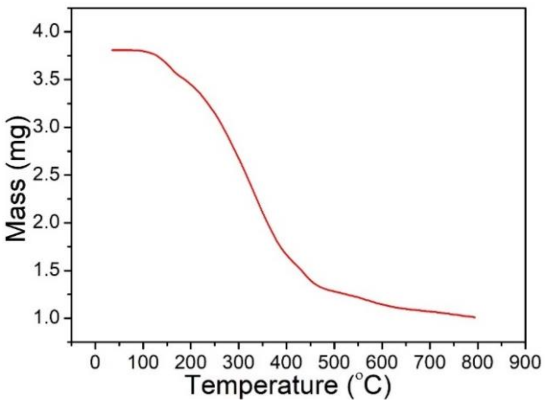

Figure S3. TGA of **CAR** under N_2, heating rate = 10 °C/min.

4. X-ray crystallography

Yellow single crystals of **CARH** were grown by allowing evaporation of a nearly saturated solution of **CARH** in DCM at room temperature. Also, black single crystals of **CAR** were grown by allowing evaporation of a nearly saturated solution of **CAR** in DCM at room temperature under N_2 condition.

The single structure of **CAR** was measured by XRD at 130, 200, 250, 290 and 340 K, respectively. For the single crystal data at 130 and 200 K, no additional geometric and ADP restraints were applied in the structure refinement. For the single crystal data at 250 and 290 K, an overall ADP restraints were used during the refinement. Removing them do not affect the structure. For the single crystal at 340 K, the N atom ellipsoid seems strange compared to surrounding atoms, due to somewhat disorder. And EDAP constraint was used to make the ADP looks more reasonable. Remove them will make N=N bonds a little shorter.

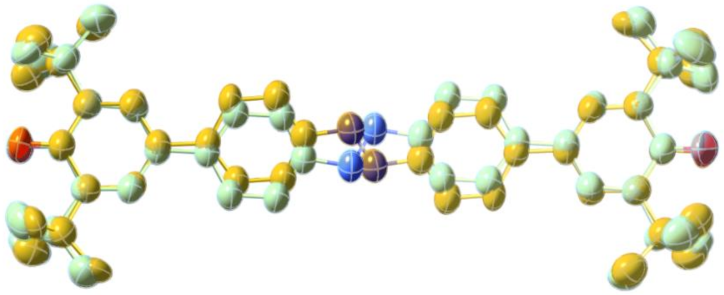

Figure S4. The major and minor conformers of disordered crystal structure of **CAR**.

Owing to the influence of temperature and molecular motion, the result of X-Ray diffraction show the appearance of disorder. For the single crystal data of 130 and 200 K, the influence of disorder is

negligible and disorder operation would enlarge the error. For the single crystal data of 250, 290 and 340 K, the results after disorder operation showed two conformations (Figure S4) and only one conformer is dominating. With the temperature increasing from 250 to 340 K, the radio of two conformer populations is constantly changing: 0.903:0.097 (250 K), 0.845:0.155 (290 K), 0.827:0.173 (340 K). The results indicate that a conformational interconversion takes place in the crystal of **CAR**. Therefore, this temperature dependence of populations of the conformers is a good proof of exist of the pedal motion in **CAR**. In addition, in spite of the presence of the disorder, the population of the minor conformer is very small all along the whole temperature range analyzed even if its ratio is rising with temperature increasing. The very little influence of the disorder on bonds length is mainly existing in the NN bond, but for the other bonds, this is negligible according to the literature report by: Harada, J.; Ogawa, K.; Tomoda, S. Molecular motion and conformational interconversion of azobenzene in crystals as studied by X-ray diffraction. *Acta Cryst.* **1997**, B53, 662-672.

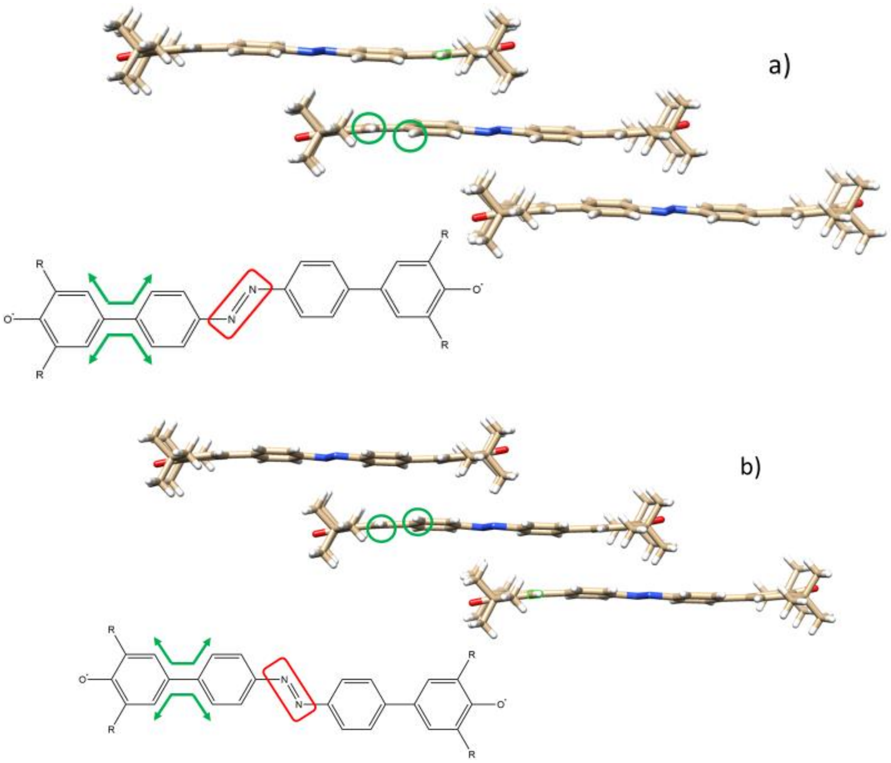

Figure S5. Portion of the crystal of **CAR** (at 340 K) showing that the positive (a) /negative (b) θ angle (sign determined by the position of the two H atoms circled in green, with respect to the molecular plane) occurs in combination with a specific orientation of the NN azo group, as schematically indicated also on the bottom left part of the figure. The same situation is observed for crystal structures at different temperatures.

In the crystal environment (unlike in the isolated molecule) the positive/negative values of the θ angle (namely the orientation of the phenoxyl group with respect to the azobenzene) is accompanied by a specific orientation of the azo group as shown in Figure S5. This is due to the presence of the two neighbor molecules above and below in the crystal environment and suggests that a sign change of the θ twisting angle can occur if accompanied by the bicycle pedal motion which rotates the azo group.

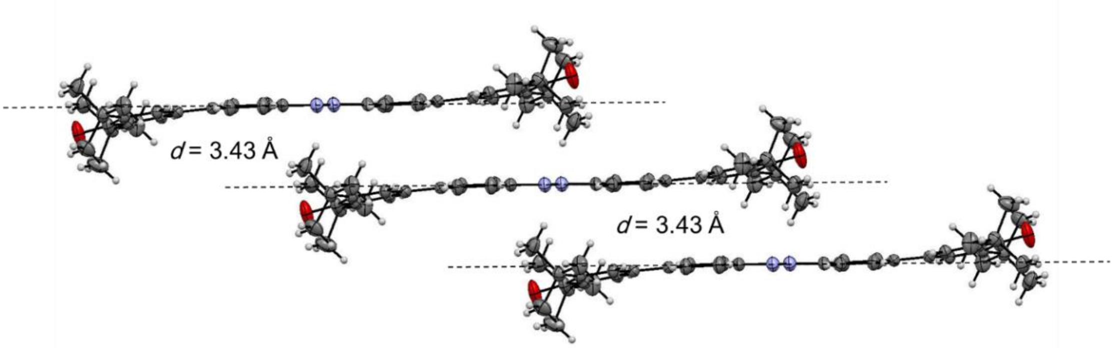

Figure S6. Partial solid-state packing diagram of **CAR** at 290 K

Table S1. Crystal data and structure refinement for **CARH**. (CCDC: 2006244)

Identification	**CARH**
Chemical formula	$C_{40}H_{50}N_2O_2$
Formula weight	590.82
Temperature	172 K
Wavelength (Å)	1.54178
Space group	P -1
Hall group	-P 1
$\mu(mm^{-1})$	0.529
a (Å)	5.9595 (4)
b (Å)	12.0514 (9)
c (Å)	13.4583 (10)
α (°)	66.982 (3)
β (°)	84.743 (3)
γ (°)	76.761 (3)
V ($Å^3$)	865.98 (11)
Z	1
ρ (g/cm^{-3})	1.133
F_{000}	320
R1	0.0435 (2750)
wR2 (all Data)	0.1253 (3033)

Table S2. Crystal data and structure refinement for **CAR** at 130 K. (CCDC: 2006243)

Identification	**CAR**
Chemical formula	$C_{40}H_{48}N_2O_2$
Formula weight	588.80
Temperature	130 K
Wavelength (Å)	1.54178
Space group	P 21/n
Hall group	-P 2yn
$\mu(mm^{-1})$	0.540
a (Å)	9.3523 (16)
b (Å)	18.579 (3)
c (Å)	9.791 (2)
α (°)	90
β (°)	94.782 (12)
γ (°)	90
V (Å^3)	1695.3 (5)
Z	2
ρ (g/cm^{-3})	1.153
F_{000}	636.0
R1	0.0817 (1855)
wR2 (all Data)	0.2515 (2962)

Table S3. Crystal data and structure refinement for **CAR** at 200 K. (CCDC: 2006239)

Identification	**CAR**
Chemical formula	$C_{40}H_{48}N_2O_2$
Formula weight	588.80
Temperature	200 K
Wavelength (Å)	0.71073
Space group	P 21/n
Hall group	-P 2yn
$\mu(mm^{-1})$	0.069
a (Å)	9.3924 (14)
b (Å)	18.590 (3)
c (Å)	9.8242 (8)
α (°)	90
β (°)	94.277 (4)
γ (°)	90
V ($Å^3$)	1710.6 (4)
Z	2
ρ (g/cm^{-3})	1.143
F_{000}	636.0
R1	0.0540 (2518)
wR2 (all Data)	0.1793 (3632)

Table S4. Crystal data and structure refinement for **CAR** at 250 K. (CCDC: 2006240)

Identification	**CAR**
Chemical formula	$C_{40}H_{48}N_2O_2$
Formula weight	588.80
Temperature	250 K
Wavelength (Å)	0.71073
Space group	P 21/n
Hall group	-P 2yn
μ(mm^{-1})	0.529
a (Å)	9.4456 (2)
b (Å)	18.6220 (5)
c (Å)	9.8660 (3)
α (°)	90
β (°)	94.2185 (17)
γ (°)	90
V (Å^3)	1730.69 (8)
Z	2
ρ (g/cm^{-3})	1.130
F_{000}	636.0
R1	0.0621 (2103)
wR2 (all Data)	0.1884 (3048)

Table S5. Crystal data and structure refinement for **CAR** at 290 K. (CCDC: 2006241)

Identification	**CAR**
Chemical formula	$C_{40}H_{48}N_2O_2$
Formula weight	588.80
Temperature	290 K
Wavelength (Å)	0.71073
Space group	P 21/n
Hall group	-P 2yn
μ(mm^{-1})	0.525
a (Å)	9.4800 (5)
b (Å)	18.6505 (10)
c (Å)	9.9068 (5)
α (°)	90
β (°)	94.252 (4)
γ (°)	90
V (Å^3)	1746.77 (16)
Z	2
ρ (g/cm^{-3})	1.120
F_{000}	636.0
R1	0.0772 (2067)
wR2 (all Data)	0.2157 (3167)

Table S6. Crystal data and structure refinement for **CAR** at 340 K.   (CCDC: 2006242)

Identification	**CAR**
Chemical formula	$C_{40}H_{48}N_2O_2$
Formula weight	588.80
Temperature	340 K
Wavelength (Å)	0.71073
Space group	P 21/n
Hall group	-P 2yn
$\mu(mm^{-1})$	0.521
a (Å)	9.5158 (10)
b (Å)	18.688 (2)
c (Å)	9.8345 (11)
α (°)	90
β (°)	94.155 (7)
γ (°)	90
V (Å^3)	1760.1 (3)
Z	2
ρ (g/cm^{-3})	1.111
F_{000}	636.0
R1	0.0750 (1987)
wR2 (all Data)	0.2430 (3080)

5. Simulated and experimental powder pattern of CAR

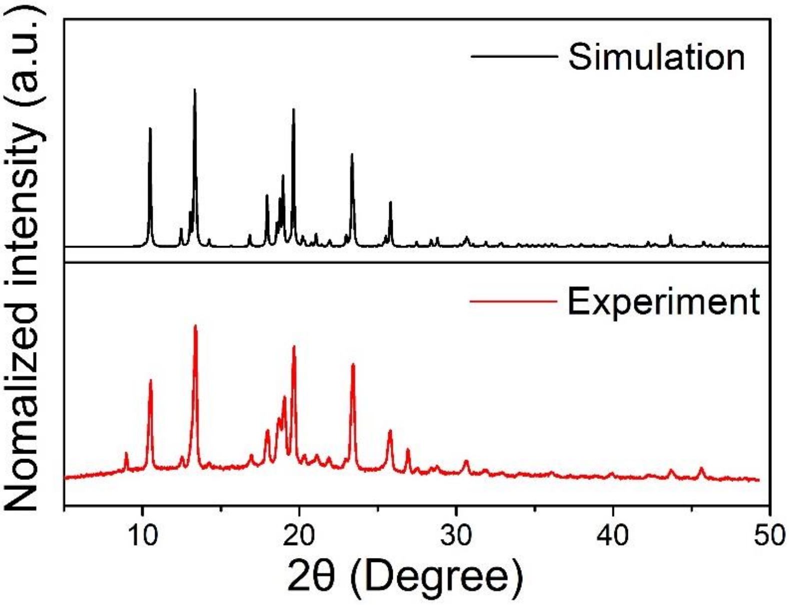

Figure S7. The simulative (black line) powder pattern based on the single crystal of 290 K and experimental (red line) powder pattern of **CAR**

The XRD of the solid powder was measured at room temperature. The result shows that the simulative powder pattern based on the single crystal of 290 K is consistent with that of the experimental result at room temperature (Figure S7). This result can indicate that the molecular stacking is consistent in crystals and powers.

6. SQUID measurements and analysis

Magnetization measurements

For SQUID measurements, first the diradical **CAR** are dried in vacuum at 50 °C for 48 hours. Magnetic susceptibility of powder samples of **CAR** (9.7 mg) was measured in a polycarbonate capsule fitted in a plastic straw as a function of temperature in heating (2 K→400 K) mode with 30 seconds of temperature stability at each temperature (3 K increment in a range 2-400 K) at 1.0 T using a SQUID magnetometer (Quantum Design MPMS-SQUID VSM-094). The data was corrected for sample diamagnetism (Pascal's constants) and the diamagnetism of the sample holder (polycarbonate capsule).

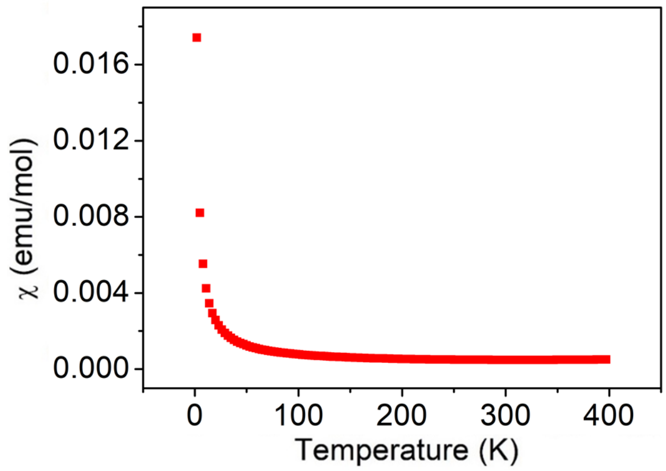

Figure S8. Temperature-dependent plots of χ for **CAR** measured at 1.0 T in the stable mode from 2 to 400 K.

Data analysis. The SQUID data in Fig. 3a were fitted with a modified Bleaney-Bowers equation.

$$\chi = \frac{Ng^2\mu_\beta^2}{kT}\left[\frac{2}{3+e^{-2J/kT}}\right](1-\rho) + \frac{Ng^2\mu_\beta^2}{2kT}\rho + TIP(1-\rho)$$

Where ρ is the fraction of s = 1/2 impurity, TIP is the temperature independent paramagnetism due to a small energy gap between group singlet state and excited triplet state.

The yield ρ = 4.9%, TIP = 9.43 x 10^{-4} emu/mol, and 2J= -3.68 kcal/mol for **CAR**, with the Adj. R-Square > 0.995.

7. ESR spectra and simulation

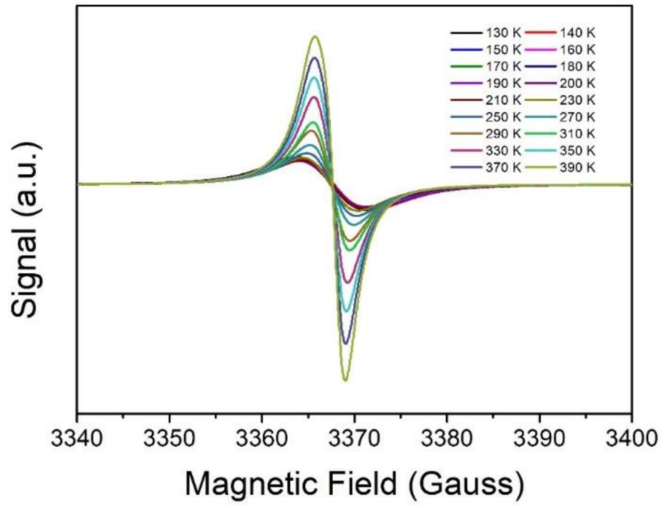

Figure S9. Variable temperature ESR spectra (130 K to 390 K) of **CAR** solid sample.

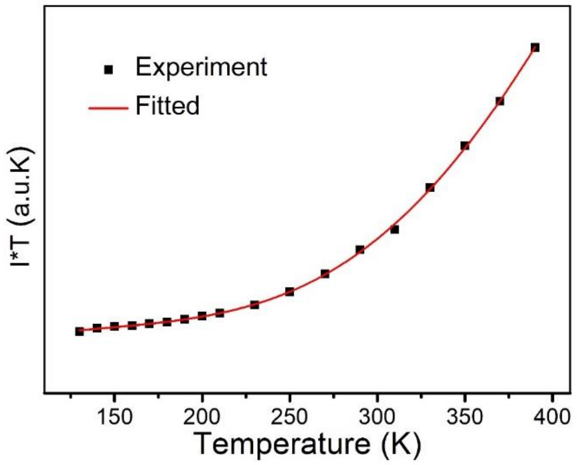

Figure S10. The measured (the black square) and fitted (the solid red line) I*T-T curves of **CAR** based on the variable temperature ESR measurements for solid (130 to 390 K). The solid line are the fitting curves according to a modified Bleaney–Bowers equation.

ESR simulation. The ESR data were also fitted with a modified Bleaney-Bowers equation. The yield ρ = 1.7%, TIP = 3.29 x 10^{-4} emu/mol, and $2J$ = -3.41 kcal/mol for **CAR**, with the Adj. R-Square > 0.995. However, there is a little difference of the results of 2J between ESR and SQUID of **CAR**. We assume that the large monoradical impurity ρ value of **CAR** is due to the intermolecular exchange interactions between adjacent molecules in the solid states. The spins between adjacent molecules are paired if the exchange interactions between two overlapping nitrogen containing rings are large enough. Then the left spin of each molecule might pair with the spin of other molecules, or stay as a doublet monoradical. Thus, there is a lot of triplet disappear with some or no increase in doublet depending on how complete the spin pairing between molecules.

To confirm this assumption the ESR signals of **CAR** in the solid state and dilute solid matrix were measured and compared. First, the ESR signal of a small amount of solid **CAR** (~ 0.5 mg) at room temperature was measured. Then excess benzophenone was added to the tube and melted to dissolve **CAR**. Followed by rapid cooling to get a solid solution, in which each radical center is surrounded by benzophenone molecule without intermolecular exchange interaction and spin pairing. Finally, the ESR spectra of the solid solution at room temperature were taken. As expected, ESR spectrum with an unresolved single peak was observed for solid **CAR**, while an enhanced triplet ESR spectrum was obtained for the solid solution. Hence, the s = 1/2 impurity (ρ) in SQUID is from intermolecular exchange interactions in the solid state.

8. UV/vis/NIR Absorption Spectrum

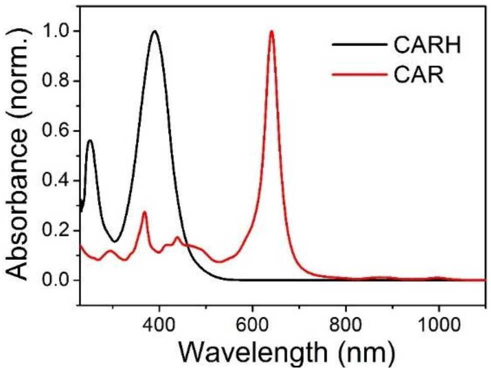

Figure S11. Normalized absorption spectra of **CARH** (black) and **CAR** (red) in DCM solution at room temperature.

9. Raman spectroscopy and theoretical Raman spectra.

The Raman spectra were recorded by using the 633 and 785 nm excitations of a Bruker Senterra Raman microscope by averaging spectra during 50 min with a resolution of 3–5 cm^{-1}. A CCD camera operating at –50 ºC was used. The spectra were collected using the 1 × 1 camera of the mentioned microscope. Variable temperature Raman spectra were obtained in a Linkam cell working up to 80 K incorporated in the microscope camera of the Senterra spectrometer.

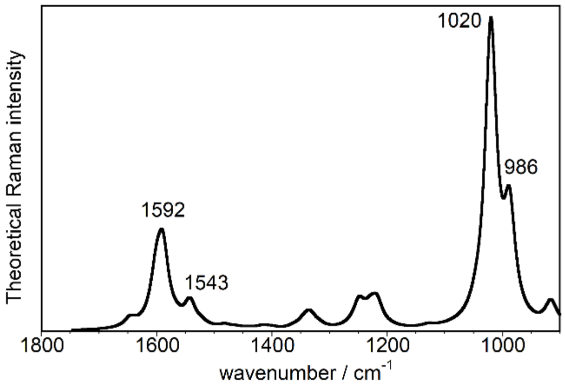

Figure S12. Theoretical Raman spectrum at the DFT/B3LYP/6-31G* level calculated on the molecular geometry of **CAR** taken from the x-ray structure at 130 K. Wavenumbers are scaled by a uniform scale factor of 0.97.

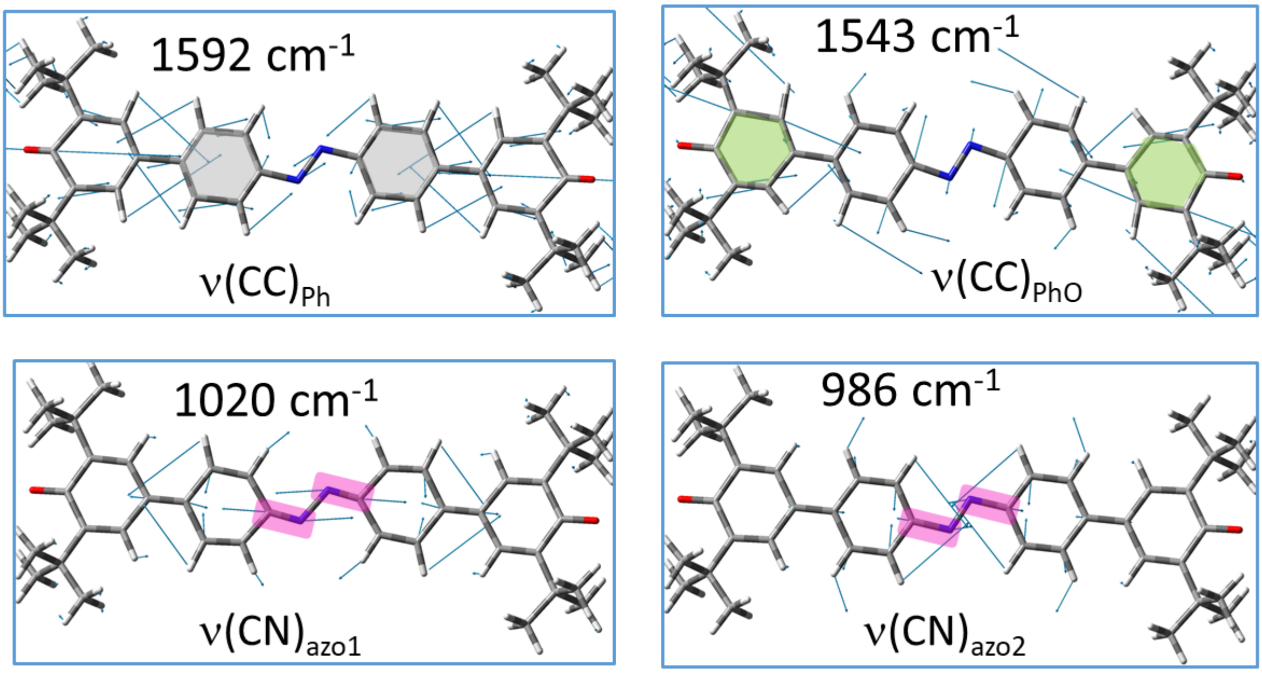

Figure S13. Vibrational normal modes associated with the theoretical wavenumbers from the spectrum in Figure S12 together with the assignments.

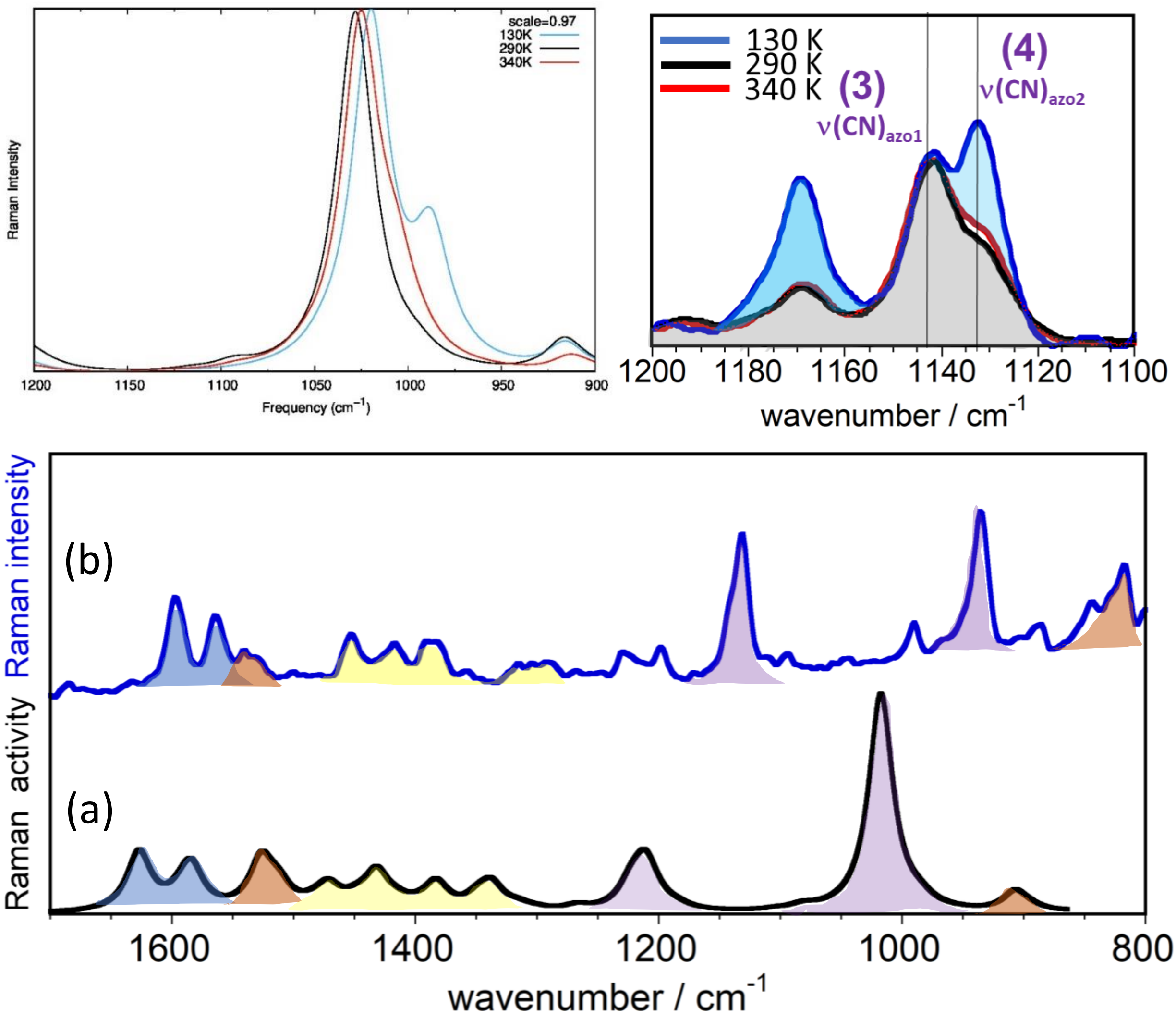

Figure S14. Top, left: Theoretical Raman spectra in the medium wavenumber region at the DFT/ B3LYP/6-31G* level calculated on the molecular geometry of **CAR** taken from the x-ray structure at 130, 290 and 340 K. Wavenumbers are scaled by a uniform scale factor of 0.97. Top, right: Experimental spectra such as in Figure 4. Bottom: Full range comparison of (a) theoretical Raman spectrum of **CAR** at the DFT/ B3LYP/6-31G* level calculated on the molecular geometry from the x-ray structure at 290 K and (b) 1064 nm FT-Raman spectrum of **CAR** at 298 K in the solid state. For the comparison of the whole spectrum the 1064 nm FT-Raman spectrum is preferred over the 633 and 785 nm since the former is in non-resonant conditions regarding the strong electronic absorption bands. On non-resonant Raman conditions. a richer vibrational Rama spectrum is obtained that better compares with the theoretical spectrum simulated in non-resonant conditions as well. Shaded band colors allow to correlate the different groups of bands between the spectra.

10. Quantum chemical and MM calculation details

We have investigated the CAR molecule as isolated molecular units and inside a portion of the crystal with DFT and QM/MM calculations. All calculations were performed with the Gaussian 16 program suite [1]. For isolated CAR molecules we used Density functional theory (DFT) with M06-2X, [2]. exchange-correlational functionals and employed the 6-311G(d) basis set [2-4]. for all atoms. Additional calculations, including Huang-Rhys parameters (see below) were carried out using B3LYP[5]. functionals and the 6-31G* basis set. Full geometry optimizations were carried out at the (U)M06-2X/6-311G(d), B3LYP/6-31G* levels, and the obtained stationary points were characterized by frequency calculations.

The spin correction for singlet-triplet energy gap was calculated using Yamaguchi's procedure which includes the energy difference between BS singlet and open-shell triplet and the correction for spin contamination:[6,7].

$$\Delta E_{\mathrm{ST}} = (E_{\mathrm{BS}} - E_{\mathrm{T}}) \frac{\langle \mathrm{S}^2 \rangle_{\mathrm{T}}}{(\langle \mathrm{S}^2 \rangle_{\mathrm{T}} - \langle \mathrm{S}^2 \rangle_{\mathrm{BS}})} \tag{1}$$

where the $\langle \mathrm{S}^2 \rangle_{\mathrm{T}}$ and $\langle \mathrm{S}^2 \rangle_{\mathrm{BS}}$ is the spin expectation values of the triplet state and broken-symmetry singlet, respectively.

The frontier molecular orbital profiles and spin densities were illustrated using Multiwfn[8] and VMD[9].

Diradical characters were determined by the calculation of different descriptors:

a) Descriptor y_0, defined as the occupation number of the lowest unoccupied natural orbital (LUNO)[10]. A molecule with $y_0 = 0$ implies a closed-shell structure, whereas a molecule with $y_0 = 1$ indicates a pure diradical structure.

b) Descriptor y_0(PUB3LYP) computed in the spin-unrestricted single-determinant formalism, with the B3LYP functional and using the spin-projection scheme as [11,12]:

$$y_0^{PUB3LYP} = 1 - \frac{2T_0}{1 + T_0^2} \tag{2}$$

with T_0 calculated as:

$$T_0 = \frac{n_{HONO} - n_{LUNO}}{2} \tag{3}$$

and n is the occupation number of the frontier natural orbitals. The values of the y_0(PUB3LYP) are generally smaller than those obtained with the approach a) but can be directly compared with those of other diradical molecules computed with the same approach.

c) The last descriptor is based on finite-temperature DFT (FT-DFT) and is the N_{FOD} value, which is the integral of the fractional orbital density (FOD) $\rho^{FOD}(r)$, over all space. The $\rho^{FOD}(r)$ is defined as [13,14]:

$$\rho^{FOD}(r) = \sum_{i}^{N}(\delta_1 - \delta_2 f_i)|\varphi_i(r)|^2 \tag{4}$$

where δ_1 and δ_2 are two constants set such that only fractionally occupied orbitals are taken into account; φ_i are molecular spin orbitals, and f_i are the fractional orbital occupancies ($0 \leq f_i \leq 1$) determined by the Fermi-Dirac distribution. In other words, the so defined FOD yields, for each point in real space, only the contribution of 'hot' or strongly correlated electrons and is therefore an analysis tool of static correlation. The NFOD parameter was computed with the ORCA 4.0.1.2 package [15] with the default setting (TPSS/def2-TZVP level with Tel=5000K).

Geometry optimization, diradical character descriptors, singlet -triplet gap.

The B3LYP/6-31G* calculations predict two different structures, one (the CS structure) substantially similar to the Q structure observed at 130K and a second one (the BS structure) which is more aromatic and more similar to the 290K observed structures. These conclusions are supported by the descriptors of diradical character (y_0(PUB3LYP) and NFOD, see Table S8) computed for the optimized geometries, and for the crystal structures at 130K, 290K and 340K. The trends for the two parameters is similar and indicates a moderate diradical character for the 130K structure, followed by the 340K structure and a larger value for the 290K structure. The CS B3LYP descriptors are closer to those of the 130K structure. The diradical descriptors computed at the BS B3LYP structure are both closer to the 290K structure.

The relative energy between the three structures Q, A and PQ were determined with Spin Flip TDDFT calculations at BHHLYP/6-31G* level. SF-TDDFT calculations were carried out in the collinear approximation as implemented in the GAMESS package [16]. Taking as reference the energy computed for the Q structure, the A structure results to be more stable than the Q structure by about 1.15 kcal/mol, while the PQ structure is computed to be more stable than the A structure by about 1.65

kcal/mol.

Table S7. Descriptors of diradical character y_0(PUB3LYP) and NFOD for the crystal structures and the computed structures of CAR.

	Diradical character descriptors	
**structure**	y0 (PUB3LYP)	NFOD
Crystal @130K (Q)	0.41	2.14
Crystal @290K (A)	0.68	2.28
Crystal @340K (PQ)	0.59	2.22
B3LYP CS	0.20	2.03
B3LYP BS	0.66	2.35

Table S8. Energy difference (kcal/mol) between the singlet Broken-Symmetry (BS) and triplet High-Spin (T) states, $(E_{BS} - E_T)$, and the corresponding corrected ΔE_{ST}, obtained at the B3LYP/6-31G* level.

	$\Delta E(singlet - triplet)$	
**structure**	$(E_{BS} - E_T)$	ΔE_{ST}
Crystal @130K (Q)	-2.62	-4.94
Crystal @290K (A)	-0.80	-1.58
Crystal @340K (PQ)	-1.20	-2.34

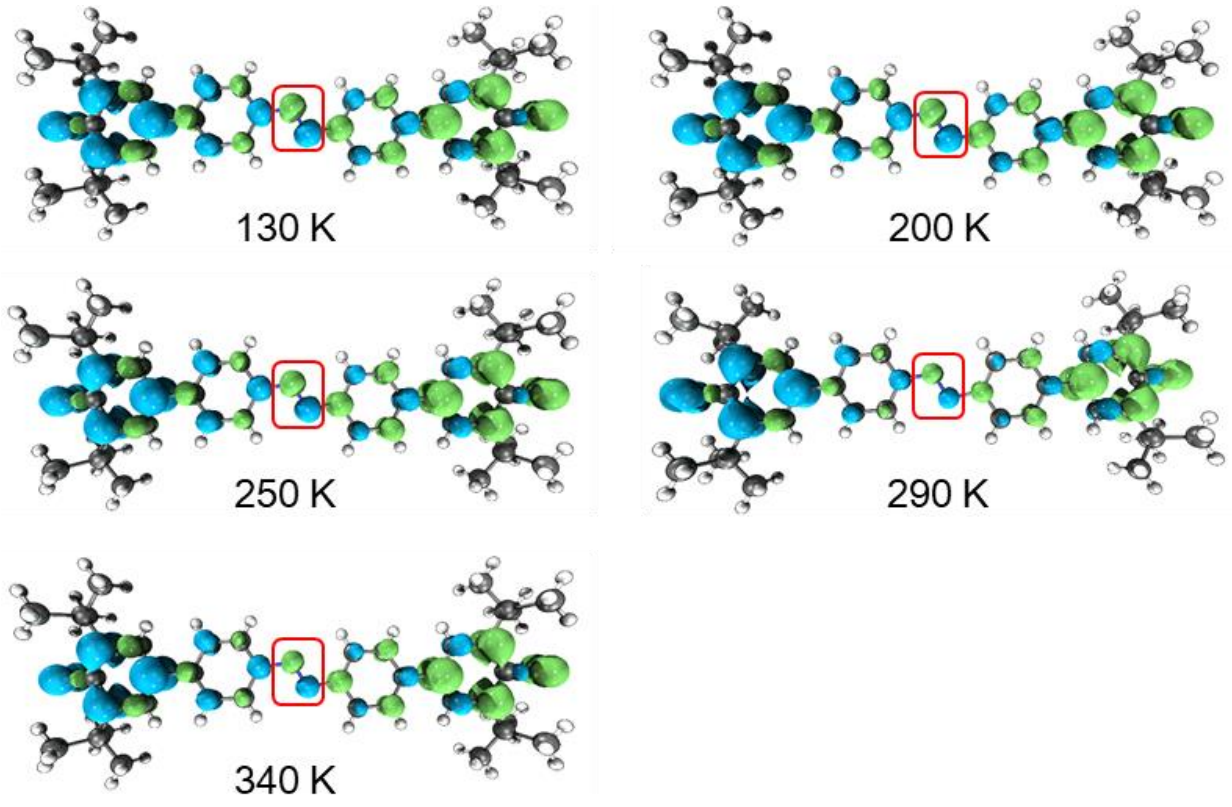

Figure S15. Calculated spin density for **CAR** based on single crystal structure at 130, 200, 250, 290

and 340 K (M06-2X/6-311g(d) level).

Table S9. Selected spin population value calculated level for **CAR** based on single crystal structure under temperature of 130, 200, 250, 290 and 340 K (M06-2X/6-311g(d) level).

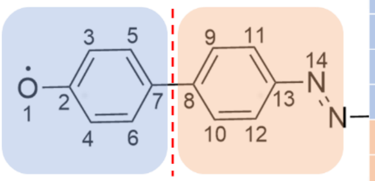

Atom	130K	200K	250K	290K	340K
1	-0.282	-0.277	-0.277	-0.312	-0.295
2	0.072	0.070	0.070	0.075	0.072
3	-0.263	-0.269	-0.268	-0.289	-0.288
4	-0.270	-0.268	-0.272	-0.292	-0.276
5	0.161	0.162	0.162	0.166	0.167
6	0.164	0.163	0.164	0.167	0.163
7	0.418	0.417	0.424	0.428	-0.427
8	0.182	0.183	0.176	0.124	0.146
9	-0.148	-0.151	-0.145	-0.106	-0.127
10	-0.135	-0.134	-0.130	-0.096	-0.114
11	0.113	0.111	0.104	0.068	0.079
12	0.115	0.112	0.105	0.067	0.082
13	-0.168	-0.168	-0.162	-0.106	-0.131
14	0.174	0.165	0.154	0.081	0.100

Huang-Rhys factors for the Q→ A and A→ PQ transformations.

The Huang-Rhys parameters [17] S_k for the Q to A and A to PQ transformations were computed for each vibrational mode *k* with frequency ν_k. Each S_k was obtained as

$$S_k = \frac{1}{2}{B_k}^2 \qquad (5)$$

where B_k is the dimensionless displacement parameter defined, assuming the harmonic approximation as:

$$B_k = \sqrt{\frac{2\pi\nu_k}{\hbar}}[\boldsymbol{X_j} - \boldsymbol{X_i}]\boldsymbol{M}^{1/2}\boldsymbol{Q}_k(j) \qquad (6)$$

where $\boldsymbol{X}_{i,j}$ is the *3N* dimensional vector of the Cartesian coordinates of the *i,j* crystal structure (here *i*=Q (130K structure),PQ (340K structure) and *j* is the A (290K structure)), $\boldsymbol{M}$ is the *3N*×*3N* diagonal matrix of atomic masses and $\boldsymbol{Q}_k(j)$ is the *3N* dimensional vector describing the *v* normal coordinate of the *j* (290K structure) state in terms of mass weighted Cartesian coordinates.

The Q to A geometry change, as for other diradical conjugated molecules, is assisted by specific vibrational normal modes, dominated by the stretching of the conjugated skeleton. To further explore

the normal modes of vibrations that assist the Q to A and A to PQ geometry change, we determined the Huang-Rhys factors Sm by projecting the geometry changes (A-Q or A-PQ) over the vibrational normal modes of the A structure, computed at B3LYP level.

The results (see Table S10) show that among high frequency modes (those dominated by stretching of the conjugated skeleton) the "Q to A" and "A to PQ" geometry changes do not display the same "Huang Rhys active" modes. This implies that the two geometry changes are not sequential along the same 'nuclear coordinate' but instead they occur along two different paths that do not necessarily overlap. This is exemplified in Table S10 by the fact that among the five high frequency vibrations shown, two are active in assisting only the "Q to A" change, two different ones are active in assisting only the "A to PQ" geometry change and only one (the 1575 cm^{-1} frequency) displays similar Huang-Rhys factors for both geometry changes. Therefore, a schematic representation of the geometry changes occurring on **CAR** crystals by increasing the temperature can hardly be represented just along a single common nuclear displacement, since it involves a multidimensional space, in which besides different stretching coordinates also the torsional coordinates may have a role.

The HR factors show that although the diradical character of the PQ structure appears to be intermediate between those of the Q and A structures, the path from A to PQ cannot be considered as simple "way back" to the Q structure along the same path, but rather as a structural evolution along a pathway which is not the same leading from the Q to the A structure.

Table S10. Computed Huang-Rhys factor (S_m) associated with the high frequency vibrational normal coordinates of **CAR**, for the two geometry changes Q-to-A and A-to-PQ.

Q-A		A-PQ
Frequency[a] /cm^{-1} (Huang-Rhys factor S_m)	Graphical representation of the normal coordinate	Frequency (cm^{-1})
	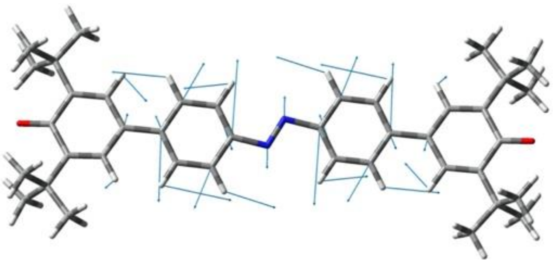	1688 (0.085)

	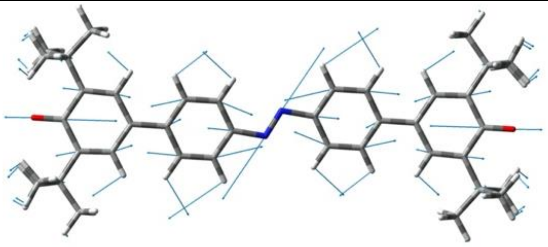	1650 (S=0.064)
1575 (0.065)	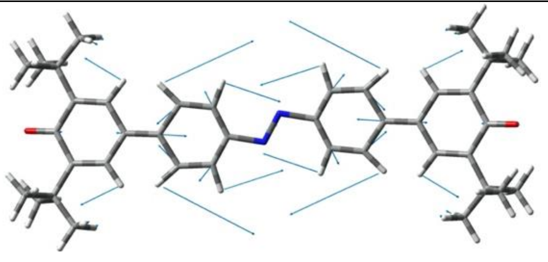	1575 (S=0.062)
1492 (0.058)	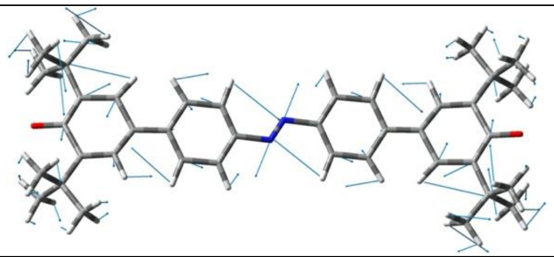	
1441 (0.081)	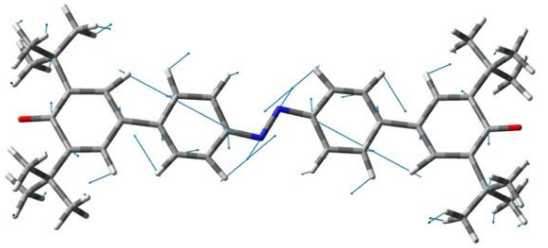	

[a]From B3LYP/6-31G* calculations, unscaled frequencies

Cluster calculations. Because the CH bond lengths of the crystal structures are unrealistically short, compared to equilibrium structures, we used the CLUSTERGEN software [18] to adjust the CH bond lengths of the **CAR** molecule in each crystal structure. We used the same software also to generate the clusters of **CAR** molecules that have been used in QM/MM calculations (see below). To explore the role of the crystal environment we computed potential energy surfaces (PES) along selected nuclear motions for a central molecule surrounded by a portion of the crystal, with QM/MM calculations. The cluster included in most cases 19 molecules (the central one surrounded by 18) but some additional calculation was done using a cluster of 47 molecules (the central one surrounded by 46). QM/MM calculations were performed with the G16 package and the ONIOM model [19], using the B3LYP functional and 6-31G* basis set for the high-level region (i.e. central **CAR** molecule) and the QM calculations included electronic embedding. The low-level region (molecular mechanics) was modelled by atomic point charges determined by the Qeq approach [20] using the Dreiding force field

[21,22] parameters attributed to the fixed molecular geometry of the surrounding CAR molecules. A picture of the 19 and 47 molecule clusters is shown in Figure S16.

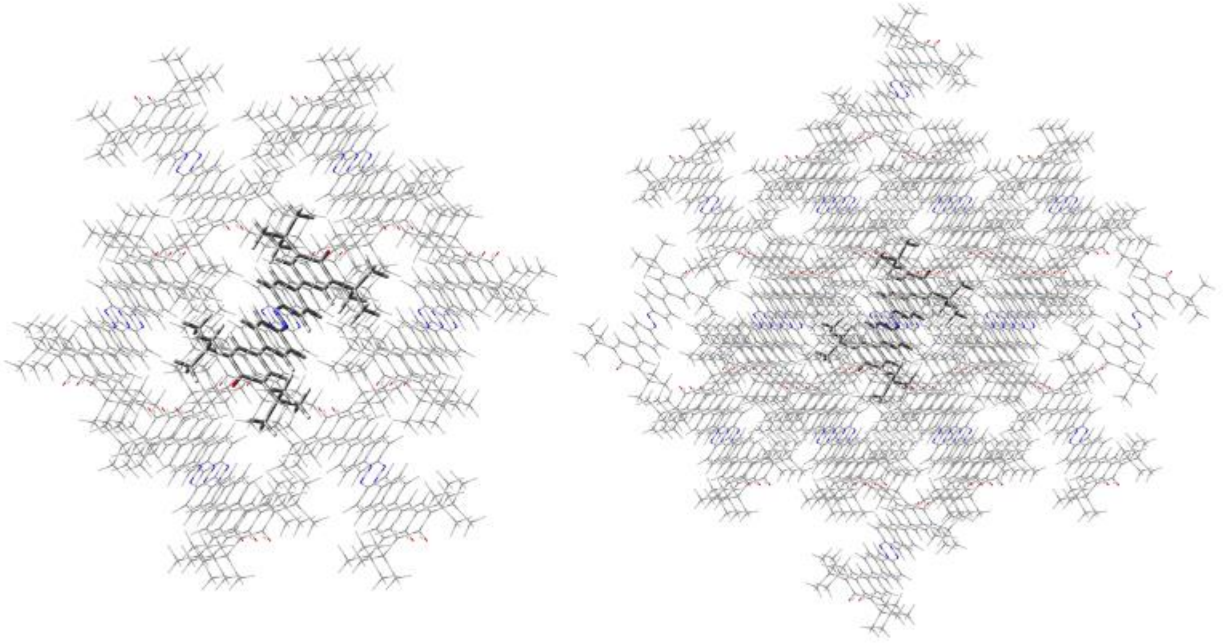

Figure S16. Representation of (left) the cluster of 19 **CAR** molecules and (right) the cluster of 47 molecules described at QM/MM level. All the molecules are taken at the crystal structure with CH bond lengths adjusted to standard values. The cluster were generated using the CLUSTERGEN software [18].

PES computed in the cluster.

The PES computed for the A and PQ structures inside the cluster and upon twist of the azobenzene with respect to the phenoxyl terminal groups is shown in Figure S17. Similarly, for the bicycle pedal motion the two PES are shown in Figure S18. The asymmetry of the PES is more marked for the twist of the azobenzene moiety and is induced by the environment. To clarify this point we did additional calculations as follows: i) the PES were computed in vacuo, ii) the PES were computed on a larger cluster including 47 molecules instead of 19; iii) the PES on the cluster of 19 molecules was determined using a different set of charges (Mulliken charges from DFT calculations rather than Qeq charges from the Dreiding force field). In all the calculations in clusters, the PES is asymmetric. The effect of changing the set of charges or increasing the size of the cluster is minor. The PES in vacuo (black curve in Figure S19) is symmetric. This indicates that the asymmetry is determined by the anisotropic environment and specifically by the presence of the two molecules, one above and one below the central molecule under inspection.

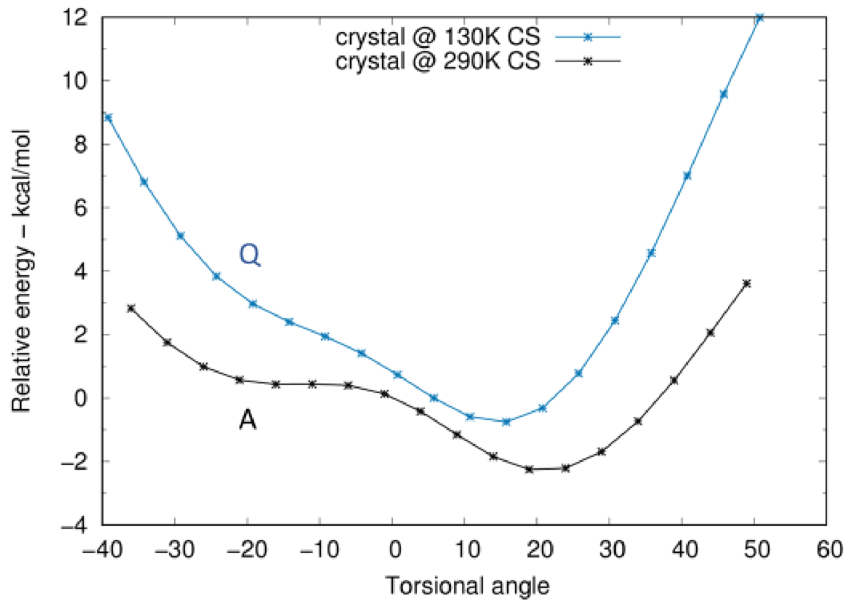

Figure S17: torsional angle potential energy profiles of the **CAR** molecule inside the crystal structure at 290 K (A form) and of that at 130 K (Q form). Calculations were carried out with the QM/MM ONIOM model on the cluster of **CAR** molecules, B3LYP/6-31G* level with electronic embedding for the central molecule, Dreiding force field and charges determined with the Qeq approach for the MM part.

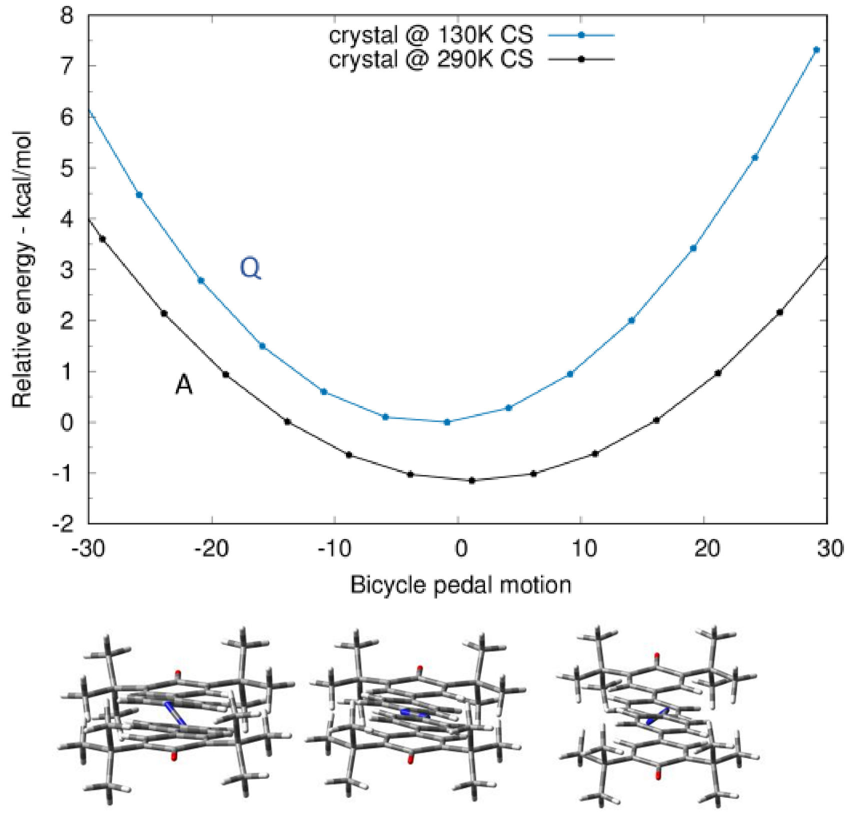

Figure S18. (top) Potential energy profiles of the **CAR** molecule inside the crystal structure at 290 K (A form) and of that at 340 K (PQ form) alongside the bicycle pedal motion distortion. (bottom) Three views of the **CAR** molecule along the bicycle pedal motion, showing the NN group oscillation during the motion. Calculations were carried out with the QM/MM ONIOM model on the cluster of **CAR** molecules, B3LYP/6-31G* level with electronic embedding for the central molecule, Dreiding force field and charges determined with the Qeq approach for the MM part.

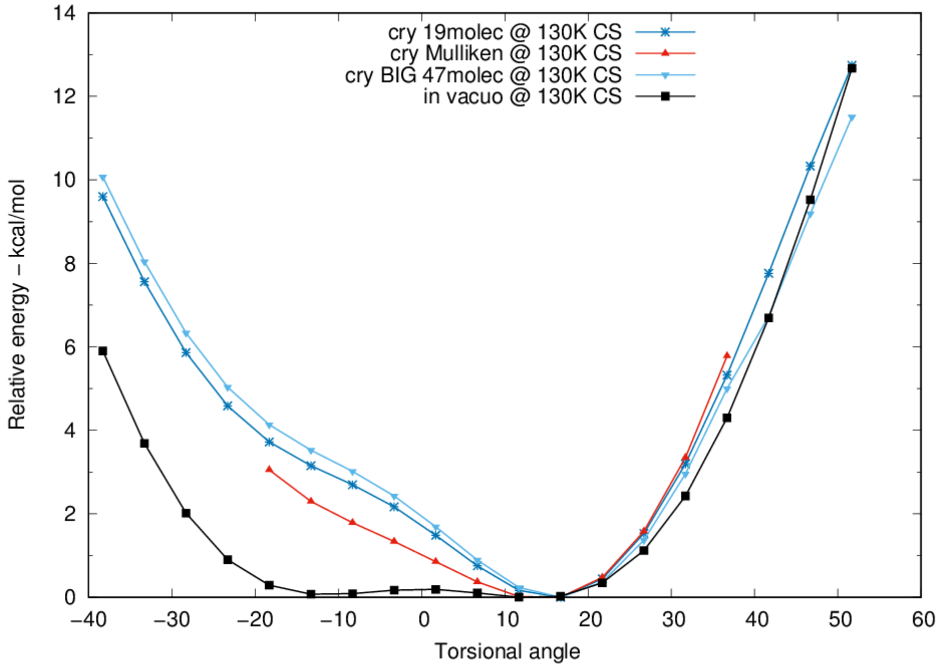

Figure S19. Comparison between torsional angle potential energy profiles of the **CAR** molecule computed with different procedures: (black) **CAR** molecule in vacuo at B3LYP/6-31G* level; (dark blue) **CAR** molecule inside a cluster of 19 molecules at the 130 K structure (Q form) QM/MM ONIOM model on the cluster of **CAR** molecules, B3LYP/6-31G* level with electronic embedding for the central molecule, Dreiding force field and charges determined with the Qeq approach for the MM part; (light blue) same as above, but in a cluster of 47 CAR molecules; (red) **CAR** molecule inside a cluster of 19 molecules at the 130 K structure (Q form) QM/MM ONIOM model on the cluster of **CAR** molecules, B3LYP/6-31G* level with electronic embedding for the central molecule, Dreiding force field and charges determined from Mulliken populations and assigned to the MM part.

11. References

[1] Frisch, M. J.; Trucks, G. W.; Schlegel, H. B.; Scuseria, G. E.; Robb, M. A.; Cheeseman, J. R.; Scalmani, G.; Barone, V.; Petersson, G. A.; Nakatsuji, H.; Li, X.; Caricato, M.; Marenich, A. V.; Bloino, J.; Janesko, B. G.; Gomperts, R.; Mennucci, B.; Hratchian, H. P.; Ortiz, J. V.; Izmaylov, A. F.; Sonnenberg, J. L.; Williams-Young, D.; Ding, F.; Lipparini, F.; Egidi, F.; Goings, J.; Peng, B.; Petrone, A.; Henderson, T.; Ranasinghe, D.; Zakrzewski, V. G.; Gao, J.; Rega, N.; Zheng, G.; Liang, W.; Hada, M.; Ehara, M.; Toyota, K.; Fukuda, R.; Hasegawa, J.; Ishida, M.; Nakajima, T.; Honda, Y.; Kitao, O.; Nakai, H.; Vreven, T.; Throssell, K.; Montgomery, J. A., Jr.; Peralta, J. E.; Ogliaro, F.; Bearpark, M. J.; Heyd, J. J.; Brothers, E. N.; Kudin, K. N.; Staroverov, V. N.; Keith, T. A.; Kobayashi, R.; Normand, J.; Raghavachari, K.; Rendell, A. P.; Burant, J. C.; Iyengar, S. S.; Tomasi, J.; Cossi, M.; Millam, J. M.; Klene, M.; Adamo, C.; Cammi, R.; Ochterski, J. W.; Martin, R. L.; Morokuma, K.; Farkas, O.; Foresman, J. B.; Fox, D. J. 2016. Gaussian 16, Revision C.01.

[2] Zhao, Y.; Truhlar, D. G. The M06 suite of density functionals for main group thermochemistry, thermochemical kinetics, noncovalent interactions, excited states, and transition elements: two new functionals and systematic testing of four M06 functionals and 12other functionals. *Theor. Chem. Acc.* **2008**, 119, 525-525.

[3] Francl, M. M.; Pietro, W. J.; Hehre, J. Self-consistent molecular orbital methods. XXIII. A polarization-type

basis set for second-row elements. *J. Chem. Phys*.**1982**, 77, 3654-3665.

[4] Frisch, M. J.; Pople, J. A.; Binkley, J. S. Self-consistent molecular orbital methods 25. Supplementary functions for Gaussian basis sets. *J. Chem. Phys*. **1984**, 80, 3265-3269.

[5] Lee, C.; Yang, W.; Parr, R. G. Development of the Colle-Salvetti correlation-energy formula into a functional of the electron density. *Phys. Rev. B*. **1988**, 37, 785.

[6] Becke, A. D. Density-functional thermochemistry. III. The role of exact exchange, *J. Chem. Phys*. **1993**, 98, 5648.

[7] Yamaguchi, K.; Fukui, H.; Fueno, T. Molecular orbital (mo) theory for magnetically interacting organic compounds. ab-initio mo calculations of the effective exchange integrals for cyclophane-type carbene dimers *Chem. Lett*. **1986**, 15, 625−628.

[8] Lu, T.; Chen, F. Multiwfn: a multifunctional wavefunction analyzer. *J. Comput. Chem*. **2012**, 33, 580-592.

[9] Humphrey, W.; Dalke, A.; Schulten, K. VMD: Visual molecular dynamics. *J. Mol. Graph*. **1996**, 14, 33-38.

[10] Nakano, M. Electronic Structure of Open-Shell Singlet Molecules: Diradical Character Viewpoint. *Topics in Current Chemistry*. **2017**, 375, 47-47.

[11] Nakano, M. Open-Shell-Character-Based Molecular Design Principles: Applications to Nonlinear Optics and Singlet Fission. *Chem. Rec*. **2017**, 17, 27-62.

[12] Yamaguchi, K. The Electronic Structures of Biradicals in the Unrestricted Hartree-Fock Approximation. *Chem. Phys. Lett*. **1975**, 33, 330-335.

[13] Grimme, S.; Hansen, A. A. Practicable Real-Space Measure And Visualization Of Static Electron-Correlation Effects. *Angew. Chem. Int. Ed*. **2015**, 54, 12308-12313.

[14] Bauer, C.; Hansen, A.; Grimme, S. The Fractional Occupation Number Weighted Density As A Versatile Analysis Tool For Molecules With A Complicated Electronic Structure. *Chem. Eur. J*. **2017**, 23, 6150-6164.

[15] Neese, F. The ORCA program system, Wiley Interdiscip. *Rev. Comput. Mol*. Sci. **2012**, 2, 73–78.

[16] Schmidt, M.W.; Baldridge, K.K.; Boatz, J.A.; Elbert, S.T.; Gordon, M.S.; Jensen, J.H.; Koseki, S.; Matsunaga, N.; Nguyen, K.A.; Su, S.J.; Windus, T.L.; Dupuis, M.; Montgomery, J.A. General atomic and molecular electronic structure system. *J. Comput. Chem*. **1973**, 14, 1347-1363.

[17] Negri, F.; Zgierski M. Z. Franck–Condon analysis of the S0→T1 absorption and phosphorescence spectra of biphenyl and bridged derivatives, *J. Chem. Phys.* **1992**, 97, 7124-7136.

[18] Kamiński, R.; Jarzembska, K. N.; Domagała, S. CLUSTERGEN: a program for molecular cluster generation from crystallographic data. *J. Appl. Crystallogr*. **2013**, 46, 540−543.

[19] Dapprich, S.; Komáromi, I.; Byun, K. S.; Morokuma, S.; M. J. Frisch. A New ONIOM Implementation in Gaussian 98. 1. The Calculation of Energies, Gradients and Vibrational Frequencies and Electric Field Derivatives, J. Mol. Struct. (Theochem). **1999**, 462 1-21.

[20] Rappé, A. K.; Bormann-Rochotte, L. M.; Wiser, D. C.; Hart, J. R.; Pietsch, M. A.; Casewit, C. J.; Skiff, W. M. APT: A next generation QM-based reactive force field model. *Mol. Phys*. **2007**, 105, 301.

[21] Mayo, S. L.; Olafson, B. D.; Goddard, W. A. III, Dreiding – A generic force-field for molecular simulations. *J. Phys. Chem*. **1990**, 94, 8897-909.

12. Cartesian coordinates for optimized geometries (M06-2X/6-311G(d))

CAR-OS

C	-9.80885500	-2.50838600	1.43279500
H	-10.36503500	-3.44656500	1.51212800
H	-10.51813900	-1.68390500	1.44420500
H	-9.16039600	-2.42758300	2.30942500
C	-8.97441300	-2.51503600	0.13804800
C	-8.14385800	-1.23831100	0.04836300
C	-6.77454600	-1.25695100	-0.00062500
H	-6.24522800	-2.19947100	0.05065100
C	-4.53661000	-0.14881300	-0.10532100
C	-3.88778400	-1.20882700	-0.75420100
H	-4.47160300	-1.96872800	-1.26039500
C	-2.50495600	-1.27051700	-0.79648500
H	-1.99288400	-2.07674400	-1.30853800
C	-1.73701600	-0.28239300	-0.18311000
C	-2.36736700	0.78003500	0.47392700
H	-1.76043000	1.53473900	0.95650500
C	-3.74621100	0.84346100	0.50412900
H	-4.22748500	1.65387600	1.03960900
C	-6.65422300	1.17927500	-0.08045600
H	-6.03736900	2.06433900	-0.16554500
C	-8.01904800	1.29806800	-0.04761100
C	-8.72164300	2.65169100	-0.09159500
C	-7.71857000	3.80797900	-0.17235200
H	-8.26483900	4.75310200	-0.19780500
H	-7.10682100	3.75901900	-1.07706100
H	-7.05522000	3.83702500	0.69639800
C	-9.63182500	2.73064600	-1.33178400
H	-10.09626400	3.71948300	-1.37968000
H	-10.41855100	1.98038800	-1.29572300
H	-9.05048700	2.58830800	-2.24682900
C	-9.56003400	2.84542200	1.18637900
H	-10.02202300	3.83646800	1.17149200
H	-8.92752000	2.78346800	2.07616300
H	-10.34641100	2.09802900	1.26300600
C	-9.90583900	-2.61809900	-1.08482100
H	-9.32686100	-2.61625700	-2.01240700
H	-10.61612900	-1.79497900	-1.11359400
H	-10.46338200	-3.55770800	-1.03905700
C	-8.08898900	-3.76613600	0.15809600
H	-7.48631100	-3.85661000	-0.74976000

H	-8.72451300	-4.65205800	0.21775700
H	-7.42121200	-3.78351300	1.02349200
C	-8.83120000	0.06767700	0.02352700
C	-6.00272100	-0.07464300	-0.06166800
N	-0.33226000	-0.44458900	-0.28061900
O	-10.06769500	0.13015400	0.06220700
C	9.80883400	2.50840500	-1.43280700
H	10.36501200	3.44658600	-1.51213600
H	10.51812000	1.68392500	-1.44423700
H	9.16036300	2.42761100	-2.30942900
C	8.97441000	2.51503800	-0.13804900
C	8.14385600	1.23831200	-0.04836700
C	6.77454400	1.25695100	0.00062200
H	6.24522600	2.19947000	-0.05065200
C	4.53661000	0.14881000	0.10531900
C	3.88778300	1.20882500	0.75420100
H	4.47160200	1.96872600	1.26039400
C	2.50495600	1.27051300	0.79648500
H	1.99288300	2.07674000	1.30853800
C	1.73701600	0.28238900	0.18311000
C	2.36736700	-0.78003900	-0.47392700
H	1.76043000	-1.53474200	-0.95650500
C	3.74621100	-0.84346400	-0.50413000
H	4.22748500	-1.65387800	-1.03961000
C	6.65422400	-1.17927600	0.08045300
H	6.03737100	-2.06434000	0.16554300
C	8.01904900	-1.29806700	0.04760600
C	8.72164600	-2.65168900	0.09159400
C	7.71857500	-3.80797500	0.17241500
H	8.26484500	-4.75309800	0.19786600
H	7.10686300	-3.75899700	1.07714800
H	7.05519100	-3.83703900	-0.69630800
C	9.63187400	-2.73061400	1.33175100
H	10.09631800	-3.71944900	1.37965100
H	10.41859600	-1.98035400	1.29564400
H	9.05057000	-2.58825700	2.24681400
C	9.55998900	-2.84545100	-1.18640700
H	10.02197400	-3.83649900	-1.17151500
H	8.92744200	-2.78351400	-2.07616900
H	10.34636600	-2.09806300	-1.26308000
C	9.90585300	2.61808500	1.08480900
H	9.32688900	2.61623200	2.01240300
H	10.61614300	1.79496400	1.11356200
H	10.46339800	3.55769300	1.03904900

C	8.08898700	3.76613800	-0.15806900
H	7.48632200	3.85660200	0.74979800
H	8.72451000	4.65206100	-0.21772800
H	7.42119700	3.78352500	-1.02345300
C	8.83120000	-0.06767500	-0.02353100
C	6.00272100	0.07464200	0.06166500
N	0.33226000	0.44458400	0.28062000
O	10.06769500	-0.13015000	-0.06219500

CAR-T

C	0.35930800	-4.15070300	9.33846100
H	1.02810400	-4.84741700	9.85140800
H	-0.48318000	-3.93537700	9.99186500
H	0.91247700	-3.22465200	9.15972100
C	-0.10518900	-4.77196600	8.00761900
C	-0.98692500	-3.78212500	7.25233500
C	-0.65693700	-3.30626200	6.00980700
H	0.26925800	-3.61290000	5.54130000
C	-1.06073800	-1.88923500	3.99007800
C	-0.39688200	-2.73119600	3.08831500
H	-0.20225700	-3.76272300	3.35814300
C	-0.02669500	-2.27111300	1.83483400
H	0.47382800	-2.91733800	1.12327500
C	-0.30164700	-0.95852100	1.45844300
C	-0.95865200	-0.10245100	2.34782600
H	-1.15612700	0.91818200	2.04752800
C	-1.33507700	-0.56832200	3.59253400
H	-1.81896900	0.10795200	4.28814800
C	-2.65606900	-1.91018600	5.91469000
H	-3.26814100	-1.22426000	5.34352500
C	-3.07463300	-2.33757700	7.14830600
C	-4.37591400	-1.85422500	7.78128400
C	-5.11178100	-0.86097100	6.87492000
H	-6.03122100	-0.54050900	7.36913900
H	-5.39329600	-1.30838600	5.91800600
H	-4.51615400	0.03491100	6.67950700
C	-5.31458700	-3.05021100	8.02884800
H	-6.26155600	-2.69027000	8.44052500
H	-4.87926500	-3.75835100	8.73033600
H	-5.53212800	-3.57040400	7.09205200
C	-4.07402700	-1.14110700	9.11321800
H	-5.00487500	-0.75599900	9.53868100
H	-3.40204500	-0.29340100	8.95358100
H	-3.61875900	-1.81859400	9.83205300

C	-0.89184800	-6.06814100	8.28122500
H	-1.23432600	-6.51696500	7.34479000
H	-1.75462300	-5.88257000	8.91692600
H	-0.24003800	-6.79025000	8.78074300
C	1.14508600	-5.14480600	7.20299400
H	0.89329700	-5.62267800	6.25230000
H	1.74074300	-5.85634000	7.77851000
H	1.77782200	-4.27663000	7.00042200
C	-2.23549000	-3.30678100	7.87876400
C	-1.46207200	-2.37357300	5.31937900
N	0.12508200	-0.58814900	0.15571300
O	-2.57316200	-3.71432400	8.99930900
C	-0.35930800	4.15070300	-9.33846100
H	-1.02810400	4.84741700	-9.85140800
H	0.48318000	3.93537700	-9.99186500
H	-0.91247700	3.22465200	-9.15972100
C	0.10518900	4.77196600	-8.00761900
C	0.98692500	3.78212500	-7.25233500
C	0.65693700	3.30626200	-6.00980700
H	-0.26925800	3.61290000	-5.54130000
C	1.06073800	1.88923500	-3.99007800
C	0.39688200	2.73119600	-3.08831500
H	0.20225700	3.76272300	-3.35814300
C	0.02669500	2.27111300	-1.83483400
H	-0.47382800	2.91733800	-1.12327500
C	0.30164700	0.95852100	-1.45844300
C	0.95865200	0.10245100	-2.34782600
H	1.15612700	-0.91818200	-2.04752800
C	1.33507700	0.56832200	-3.59253400
H	1.81896900	-0.10795200	-4.28814800
C	2.65606900	1.91018600	-5.91469000
H	3.26814100	1.22426000	-5.34352500
C	3.07463300	2.33757700	-7.14830600
C	4.37591400	1.85422500	-7.78128400
C	5.11178100	0.86097100	-6.87492000
H	6.03122100	0.54050900	-7.36913900
H	5.39329600	1.30838600	-5.91800600
H	4.51615400	-0.03491100	-6.67950700
C	5.31458700	3.05021100	-8.02884800
H	6.26155600	2.69027000	-8.44052500
H	4.87926500	3.75835100	-8.73033600
H	5.53212800	3.57040400	-7.09205200
C	4.07402700	1.14110700	-9.11321800
H	5.00487500	0.75599900	-9.53868100

H	3.40204500	0.29340100	-8.95358100
H	3.61875900	1.81859400	-9.83205300
C	0.89184800	6.06814100	-8.28122500
H	1.23432600	6.51696500	-7.34479000
H	1.75462300	5.88257000	-8.91692600
H	0.24003800	6.79025000	-8.78074300
C	-1.14508600	5.14480600	-7.20299400
H	-0.89329700	5.62267800	-6.25230000
H	-1.74074300	5.85634000	-7.77851000
H	-1.77782200	4.27663000	-7.00042200
C	2.23549000	3.30678100	-7.87876400
C	1.46207200	2.37357300	-5.31937900
N	-0.12508200	0.58814900	-0.15571300
O	2.57316200	3.71432400	-8.99930900

CAR-CS

C	0.31774400	-4.31093100	9.29919300
H	0.94063000	-5.05643000	9.80124400
H	-0.52023100	-4.06952700	9.94926200
H	0.92194500	-3.41112400	9.15486800
C	-0.15875200	-4.86812700	7.94474500
C	-0.97663300	-3.81051700	7.20639300
C	-0.61538800	-3.32073400	6.00165000
H	0.28813800	-3.69614300	5.54792200
C	-0.95570900	-1.84344000	4.03852600
C	0.25690600	-2.31056700	3.39255100
H	0.87256000	-3.05475700	3.87681700
C	0.65572300	-1.84454800	2.19098300
H	1.56434600	-2.20258700	1.72142400
C	-0.10249200	-0.84989800	1.47266700
C	-1.31597300	-0.37063500	2.09819500
H	-1.89718800	0.37442000	1.57363900
C	-1.70663700	-0.84228000	3.30011800
H	-2.62183600	-0.44809200	3.71848200
C	-2.58163100	-1.84023700	5.91347900
H	-3.16358900	-1.09546000	5.39428600
C	-3.02108600	-2.27048700	7.11503100
C	-4.30243500	-1.74586300	7.75981500
C	-4.99338500	-0.70090500	6.87689900
H	-5.90172600	-0.35663500	7.37548800
H	-5.28903800	-1.11305100	5.90809000
H	-4.36191900	0.17566400	6.70743000
C	-5.29310200	-2.90512200	7.97605200
H	-6.22496000	-2.51483900	8.39449500

H	-4.89417600	-3.64943000	8.66154500
H	-5.52995600	-3.39230700	7.02626500
C	-3.97546300	-1.07736800	9.10825800
H	-4.89000200	-0.66152700	9.54011000
H	-3.26648300	-0.25654900	8.96985500
H	-3.55425500	-1.78835500	9.81541800
C	-1.01301700	-6.13007100	8.16817900
H	-1.36344600	-6.53250200	7.21380100
H	-1.87488600	-5.92317300	8.79860400
H	-0.40657100	-6.89930400	8.65426300
C	1.08317000	-5.28129200	7.14745800
H	0.82254600	-5.72113900	6.18078500
H	1.63447300	-6.03663600	7.71115300
H	1.76155600	-4.44028900	6.97902900
C	-2.22493000	-3.29978200	7.84610300
C	-1.37157100	-2.31956500	5.27900500
N	0.37340900	-0.46329200	0.31094000
O	-2.58458400	-3.71291300	8.93706900
C	-0.31774400	4.31093100	-9.29919300
H	-0.94063000	5.05643000	-9.80124400
H	0.52023100	4.06952700	-9.94926200
H	-0.92194500	3.41112400	-9.15486800
C	0.15875200	4.86812700	-7.94474500
C	0.97663300	3.81051700	-7.20639300
C	0.61538800	3.32073400	-6.00165000
H	-0.28813800	3.69614300	-5.54792200
C	0.95570900	1.84344000	-4.03852600
C	-0.25690600	2.31056700	-3.39255100
H	-0.87256000	3.05475700	-3.87681700
C	-0.65572300	1.84454800	-2.19098300
H	-1.56434600	2.20258700	-1.72142400
C	0.10249200	0.84989800	-1.47266700
C	1.31597300	0.37063500	-2.09819500
H	1.89718800	-0.37442000	-1.57363900
C	1.70663700	0.84228000	-3.30011800
H	2.62183600	0.44809200	-3.71848200
C	2.58163100	1.84023700	-5.91347900
H	3.16358900	1.09546000	-5.39428600
C	3.02108600	2.27048700	-7.11503100
C	4.30243500	1.74586300	-7.75981500
C	4.99338500	0.70090500	-6.87689900
H	5.90172600	0.35663500	-7.37548800
H	5.28903800	1.11305100	-5.90809000
H	4.36191900	-0.17566400	-6.70743000

C	5.29310200	2.90512200	-7.97605200
H	6.22496000	2.51483900	-8.39449500
H	4.89417600	3.64943000	-8.66154500
H	5.52995600	3.39230700	-7.02626500
C	3.97546300	1.07736800	-9.10825800
H	4.89000200	0.66152700	-9.54011000
H	3.26648300	0.25654900	-8.96985500
H	3.55425500	1.78835500	-9.81541800
C	1.01301700	6.13007100	-8.16817900
H	1.36344600	6.53250200	-7.21380100
H	1.87488600	5.92317300	-8.79860400
H	0.40657100	6.89930400	-8.65426300
C	-1.08317000	5.28129200	-7.14745800
H	-0.82254600	5.72113900	-6.18078500
H	-1.63447300	6.03663600	-7.71115300
H	-1.76155600	4.44028900	-6.97902900
C	2.22493000	3.29978200	-7.84610300
C	1.37157100	2.31956500	-5.27900500
N	-0.37340900	0.46329200	-0.31094000
O	2.58458400	3.71291300	-8.93706900

13. Cartesian coordinates for optimized geometries (B3LYP/6-31G*)

CAR-CS

O	2.656840	3.733394	-8.992662
N	-0.292731	0.531699	-0.257207
C	0.180422	0.901803	-1.467530
C	-0.424893	2.049643	-2.063911
H	-1.208559	2.549619	-1.503237
C	-0.028927	2.509441	-3.288916
H	-0.503506	3.400386	-3.681390
C	1.023441	1.874392	-4.035190
C	1.226000	0.248494	-2.197943
H	1.682069	-0.631475	-1.760746
C	1.625841	0.721331	-3.414948
H	2.401382	0.178968	-3.942477
C	1.444527	2.352291	-5.308460
C	1.043558	3.827668	-7.249016
C	0.688863	3.344750	-6.023644
H	-0.231091	3.694435	-5.577632
C	2.292192	3.318167	-7.881455
C	3.092396	2.298340	-7.148118
C	2.643384	1.865038	-5.935064

H	3.238277	1.144785	-5.392148
C	0.195839	4.868551	-7.997092
C	-1.058266	5.274883	-7.197654
H	-1.623363	6.018532	-7.769880
H	-0.804525	5.729488	-6.232466
H	-1.727451	4.425370	-7.016032
C	-0.279032	4.287540	-9.353622
H	-0.893848	5.029838	-9.877159
H	-0.893524	3.392113	-9.199884
H	0.567093	4.026028	-9.990010
C	1.032932	6.150616	-8.241372
H	1.913500	5.938111	-8.848487
H	1.358874	6.589950	-7.290686
H	0.420217	6.896380	-8.762466
C	4.394574	1.775744	-7.775067
C	5.093014	0.736691	-6.875291
H	4.467713	-0.147178	-6.700908
H	5.379899	1.155980	-5.903503
H	6.010412	0.395175	-7.366752
C	4.086568	1.091208	-9.131771
H	3.634858	1.792055	-9.834601
H	3.405049	0.242916	-8.994040
H	5.015975	0.706644	-9.569322
C	5.384139	2.948817	-7.993937
H	6.315472	2.568231	-8.430815
H	5.633735	3.430694	-7.040776
H	4.966148	3.698912	-8.666195
O	-2.656840	-3.733394	8.992662
N	0.292731	-0.531699	0.257207
C	-0.180422	-0.901803	1.467530
C	0.424893	-2.049643	2.063911
H	1.208559	-2.549619	1.503237
C	0.028927	-2.509441	3.288916
H	0.503506	-3.400386	3.681390
C	-1.023441	-1.874392	4.035190
C	-1.226000	-0.248494	2.197943
H	-1.682069	0.631475	1.760746
C	-1.625841	-0.721331	3.414948
H	-2.401382	-0.178968	3.942477
C	-1.444527	-2.352291	5.308460
C	-1.043558	-3.827668	7.249016
C	-0.688863	-3.344750	6.023644
H	0.231091	-3.694435	5.577632
C	-2.292192	-3.318167	7.881455

C	-3.092396	-2.298340	7.148118
C	-2.643384	-1.865038	5.935064
H	-3.238277	-1.144785	5.392148
C	-0.195839	-4.868551	7.997092
C	1.058266	-5.274883	7.197654
H	1.623363	-6.018532	7.769880
H	0.804525	-5.729488	6.232466
H	1.727451	-4.425370	7.016032
C	0.279032	-4.287540	9.353622
H	0.893848	-5.029838	9.877159
H	0.893524	-3.392113	9.199884
H	-0.567093	-4.026028	9.990010
C	-1.032932	-6.150616	8.241372
H	-1.913500	-5.938111	8.848487
H	-1.358874	-6.589950	7.290686
H	-0.420217	-6.896380	8.762466
C	-4.394574	-1.775744	7.775067
C	-5.093014	-0.736691	6.875291
H	-4.467713	0.147178	6.700908
H	-5.379899	-1.155980	5.903503
H	-6.010412	-0.395175	7.366752
C	-4.086568	-1.091208	9.131771
H	-3.634858	-1.792055	9.834601
H	-3.405049	-0.242916	8.994040
H	-5.015975	-0.706644	9.569322
C	-5.384139	-2.948817	7.993937
H	-6.315472	-2.568231	8.430815
H	-5.633735	-3.430694	7.040776
H	-4.966148	-3.698912	8.666195

CAR-BS

O	10.122413	-0.139453	-0.044374
N	0.343667	0.477608	0.239627
C	1.734053	0.301100	0.152536
C	2.517031	1.347208	0.669722
H	2.008227	2.201561	1.105398
C	3.900832	1.280497	0.632125
H	4.480224	2.088195	1.066818
C	4.563192	0.158378	0.084307
C	2.377083	-0.824137	-0.403823
H	1.769860	-1.623034	-0.813724
C	3.757427	-0.890044	-0.429318
H	4.232739	-1.750387	-0.889460
C	6.025775	0.079684	0.047897

C	8.189255	1.239818	-0.041493
C	6.814295	1.260482	-0.000331
H	6.295118	2.209057	-0.049016
C	8.872226	-0.072665	-0.017256
C	8.054930	-1.304818	0.039579
C	6.685041	-1.178566	0.061525
H	6.070786	-2.066709	0.135466
C	9.023281	2.527887	-0.121295
C	8.136598	3.789245	-0.146104
H	8.776637	4.676561	-0.199312
H	7.523618	3.882370	0.758295
H	7.472594	3.812192	-1.018254
C	9.875772	2.526512	-1.416117
H	10.456327	3.455223	-1.475536
H	9.234848	2.474957	-2.304656
H	10.566284	1.682559	-1.433619
C	9.951936	2.630055	1.116123
H	10.643661	1.788186	1.160988
H	9.365615	2.653007	2.042810
H	10.532857	3.558997	1.064364
C	8.749235	-2.674879	0.082893
C	7.734735	-3.834484	0.146037
H	7.076151	-3.856678	-0.730388
H	7.111046	-3.791294	1.046705
H	8.277988	-4.785331	0.170293
C	9.607136	-2.869931	-1.193893
H	10.382997	-2.107245	-1.268636
H	8.982220	-2.824897	-2.094151
H	10.086257	-3.856271	-1.167901
C	9.652237	-2.770465	1.339348
H	10.132707	-3.755889	1.373274
H	9.059468	-2.655331	2.254948
H	10.428666	-2.004717	1.326875
O	-10.122413	0.139453	0.044369
N	-0.343667	-0.477608	-0.239625
C	-1.734053	-0.301100	-0.152534
C	-2.517031	-1.347208	-0.669720
H	-2.008227	-2.201561	-1.105396
C	-3.900832	-1.280498	-0.632123
H	-4.480224	-2.088195	-1.066817
C	-4.563192	-0.158378	-0.084305
C	-2.377083	0.824137	0.403825
H	-1.769860	1.623034	0.813726
C	-3.757427	0.890044	0.429320

H	-4.232739	1.750387	0.889462
C	-6.025775	-0.079684	-0.047896
C	-8.189255	-1.239818	0.041495
C	-6.814295	-1.260482	0.000332
H	-6.295118	-2.209057	0.049018
C	-8.872226	0.072665	0.017257
C	-8.054930	1.304818	-0.039578
C	-6.685040	1.178566	-0.061524
H	-6.070785	2.066709	-0.135465
C	-9.023281	-2.527887	0.121294
C	-8.136598	-3.789246	0.146091
H	-8.776638	-4.676561	0.199299
H	-7.523625	-3.882366	-0.758312
H	-7.472589	-3.812197	1.018236
C	-9.875764	-2.526519	1.416122
H	-10.456318	-3.455230	1.475540
H	-9.234834	-2.474968	2.304658
H	-10.566276	-1.682566	1.433633
C	-9.951944	-2.630047	-1.116118
H	-10.643669	-1.788177	-1.160974
H	-9.365629	-2.652995	-2.042809
H	-10.532866	-3.558989	-1.064361
C	-8.749235	2.674879	-0.082894
C	-7.734735	3.834483	-0.146050
H	-7.076145	3.856681	0.730370
H	-7.111052	3.791290	-1.046722
H	-8.277988	4.785331	-0.170307
C	-9.607128	2.869938	1.193896
H	-10.382989	2.107253	1.268648
H	-8.982205	2.824908	2.094151
H	-10.086248	3.856279	1.167903
C	-9.652245	2.770458	-1.339344
H	-10.132715	3.755883	-1.373271
H	-9.059482	2.655320	-2.254947
H	-10.428674	2.004710	-1.326862
